# ‘Whatever your job is, we are all about doing that thing super well’: High‐reliability followership as a key component of operational success in elite air force teams

**DOI:** 10.1111/bjso.12882

**Published:** 2025-04-01

**Authors:** Sally Knox, Kïrsten A. Way, S. Alexander Haslam

**Affiliations:** ^1^ School of Psychology The University of Queensland St Lucia Queensland Australia

**Keywords:** high‐reliability followership, high‐reliability organizations, identity leadership, leadership, military, social identity

## Abstract

The military is widely regarded as an extension and tool of government and society, and unreliable military behaviour during operations can have far‐reaching strategic and political consequences. Historically, literature has focused on the role of leaders in preventing disaster, emphasizing their traits, styles and attributes. Building on the Social Identity approach and High‐Reliability Organization theorizing, this paper uses thematic analysis to develop an alternative understanding of leadership as a group process to which all members contribute—not least, the front‐line personnel who do the followership that is ultimately the proof of leadership. Supported by evidence from semi‐structured interviews with air force personnel (*N =* 25), analysis points to the importance of collective mind and social identity (a shared sense of ‘us’). It also suggests that social identity strength, content and alignment—and the identity leadership shaping this—provides a basis for the *High‐Reliability Followership* that allows military groups to avoid potentially disastrous events. In this way, the creation of HROs hinges on the combined actions of identity leaders who work to represent, advance and create a specific sense of shared identity and engaged followers who internalize that identity content and enact it through behaviour that supports high reliability.

## INTRODUCTION

On a world stage where the military is seen as an extension and tool of government and the society it represents and serves, the consequences of ineffective leadership are readily apparent (e.g. Lord, 2012; Morath et al., 2011). In particular, it is clear that problematic military action can have unintended international ramifications. For just as the presence of personnel or equipment can signpost a nation's strategic political aims, so too the behaviour of individual military personnel, or groups of personnel, can be taken as a sign of a nation's values and morality (Lucas, 2007; Prentice et al., 1994). In this way, infractions by a few can undermine the legitimacy of a war or even inadvertently trigger the commencement or escalation of retaliatory military attacks (Hannah et al., 2014). Accordingly, highly reliable behaviour, shaped by effective leadership, is critically important not only because it gives militaries the edge in modern warfare but also—and sometimes more importantly—because it saves them from disaster.

Despite some notable exceptions, military behaviour is, or at least aspires to be, highly reliable (Weick & Roberts, 1993). Nevertheless, to date, most research on High‐Reliability Organizations (HROs) has concentrated on their nature rather than the processes which create and maintain them (Haslam et al., 2022). As a result, the HRO literature has had a largely descriptive focus that centres on the development of tools and procedures for assessing and cataloguing HRO practices (Haslam et al., 2022). In particular, research has centred around discussion of five hallmarks of HROs—sensitivity to operations, pre‐occupation with failure, reluctance to simplify, commitment to resilience and deference to expertise—which Weick and Sutcliffe (2015) have argued together describe aspects of high reliability in relevant organizations.

Because they represent characteristics of operations to which HROs aspire, these characteristics effectively function as guides to high‐reliability leadership. That is, they represent ‘ways of being’ that, implicitly at least, need to inform the activities and practices of leaders (Alavosius et al., 2017). What is not clear, though, is how these principles relate to what personnel do ‘on the ground’ and how their *followership* helps to realize goals of high reliability (or not).

Coupled with this, traditionally, military research has focused on leaders, and in particular the individual traits that allow them to ‘lead’ effectively. Yet the question of what followership might look like is largely neglected. This is a critical omission for two key reasons. First, it is followership—‘the characteristics, behaviours, and processes of individuals acting in relation to leaders’ (Uhl‐Bien et al., 2014, p. 96)—that provides the ultimate proof of leadership (Bennis, 1999; Platow et al., 2015), and second, followership and leadership are not simply mirror images of each other (Uhl‐Bien et al., 2014). Instead, followership is a process through which followers actively and *creatively* engage with leaders to co‐create leadership, followership and collective outcomes (Haslam et al., 2022).

As things stand, though, we have a limited understanding of how leadership and followership combine to create and maintain HROs (Epitropaki et al., 2017; Haslam et al., 2022). It is this lacuna that the present research seeks to address. It does so by exploring the nature of *high‐reliability followership* (HRF) in elite military teams. More particularly, it seeks to understand how cognitions and behaviours associated with the five HRO principles identified by Weick and Sutcliffe (2015) play out among the members of teams (i.e. followers) who are responsible for ‘doing’ high reliability on the front line.

### High‐reliability organizations (HROs)

An HRO is one in which major accidents occur infrequently even though, as a consequence of the complex environment in which the organization is operating, multiple accidents might be expected (Weick & Roberts, 1993). HRO theorizing is particularly relevant in contexts—like those often encountered in the military—which are volatile, uncertain, complex and ambiguous (VUCA) and where opportunities for direct supervision via a chain of command are often limited (Bennis & Nanus, 1985; Fraher et al., 2017).

As we noted above, in their pioneering study of HROs, Weick and Sutcliffe (2015) identified five cognitive and behavioural hallmarks of HROs (see Table 1). Importantly too, they argued that together these five hallmarks are underpinned by a sense of *collective mind* whereby the connections and relationships between individuals are instantiated in ‘collective mental functioning’ that allows the group to engage in reliable forms of collective action (Weick & Roberts, 1993; Weick & Sutcliffe, 2015, p. 22). In these terms, high reliability is a manifestation of the group's ability to *comprehend* and then *organize* itself as an entity in ways that allow its members to act consistently and reliably (Martínez‐Córcoles & Vogus, 2020; Weick & Roberts, 1993).

**TABLE 1:** Hallmark practices of HROs.

High‐reliability organization principles^a^	Entails
Reluctance to simplify	Digging below the surface to engage appropriately with the breadth and depth of matters that are critical for operational safety and/or task completion
Pre‐occupation with failure	Recognizing all failure as problematic for the organization and working to eliminate the possibility of even minor adverse events
Commitment to resilience	Being motivated to ensure that organizations are not disabled by any failures that occur and are able to recover from them quickly
Deference to expertise	Ensuring that those most qualified to provide input are able and empowered to do so
Sensitivity to operations	Having a comprehensive understanding of organizational activities and processes so as to be able to anticipate and respond to problems

^a^ These are the themes identified by Weick and Sutcliffe (2015), as defined by Haslam et al. (2022).

### Social identity

Aligned with many of the insights from work on HROs, in their seminal research on the psychology of social groups, Tajfel and Turner (1979) highlight the importance of *social identity* for group behaviour. In particular, self‐categorisation theory (Turner, 1999; Turner et al., 1987, 1994) posits that group behaviour is made possible by a cognitive redefinition of the self that leads people to understand themselves not as unique individuals (i.e. in terms of personal identity as ‘I’ and ‘me’) but rather as group members (in terms of social identity as ‘we’ and ‘us’; Turner, 1982). Military contexts are ones in which social identities abound (de Graaff et al., 2017; Jansen & Delahaij, 2020; Kylin, 2012; White et al., 2021). This is particularly evident in specialist roles within the military, where elite units and specializations each have their own distinctive, often very strong, group identities (Balkin & Schjoedt, 2012). A key reason why team members' strength of identification and connection to their group is important is because it predicts the degree to which group members are motivated to act in ways that support the group (Ellemers et al., 2004). Nevertheless, on its own, strength of social identification gives no clue as to the meaning of group members' behaviour or of the direction in which it will tend (Reutas et al., 2023). Accordingly, Reutas et al. (2023) stress that when it comes to understanding behaviour within the group it is equally important to understand what group members believe it means to be a member of the group—that is, to know about social identity *content*—and how this aligns across the group (see also Steffens et al., 2023). In the case of HROs, this analysis therefore implies that identity strength, content and alignment combine to shape high‐reliability behaviour.

Consistent with this conclusion, in their recent reflections on the psychology of HROs, Haslam et al. (2022) have pointed to the clear conceptual links between Weick and Roberts's (1993) notion of collective mind, and Tajfel and Turner's (1979) reflections on the importance of social identity for group behaviour. In particular, they argue that a group's shared social identity is the basis for a sense of collective mind and that high reliability is supported by shared social identity content that is aligned with higher order reliability‐seeking organizational goals. In this way, as individuals connect and take action as a group, their cognitions and behaviour are shaped by a shared ambition to create, and be part of, a reliability‐seeking social system (Haslam et al., 2020; Postmes et al., 2005; Weick & Sutcliffe, 2015).

Importantly, the social system that results from this self‐categorization process is not representative of any one individual but is developed through, and instantiated in, relationships and interactions with others in the group (Turner, 1999; Turner et al., 1994). This system of relationships is never ossified, ‘set’ or finalized but rather reflects a dynamic and organic sense of ‘us’ that is continually adapting to context (Haslam et al., 2020; Weick & Roberts, 1993). According to social identity theorists, it is therefore through the creation of ‘us’—and shared social identity of a particular type—that highly reliable behaviour is constructed, reinforced and repeated in the form of coordinated group action. Or, put differently, group members are understood to engage in high‐reliability behaviours because they have internalized particular social identity content as a representation of who ‘we’ are and embrace this as a manifestation of their collective mind (Haslam et al., 2022; Weick & Sutcliffe, 2015).

The present study seeks to test these ideas and to flesh out the social identity approach to HROs by exploring the experiences and beliefs of team members on the ground who are ‘doing’ high reliability. A qualitative approach was chosen to capture participants' perspectives in their own words, offering rich insights into identity‐driven behaviours that are challenging to quantify. In the process, it also expands upon a related claim of social identity researchers—namely that the forms of social identity that support high reliability are only made possible by *leadership* that builds and sustains them.

### Identity leadership

Haslam et al. (2022) argue that in order to create and maintain HROs, leaders need to engage in forms of *identity leadership* that encourage followers to embrace and enact a particular collective mind. Along these lines, previous research in military environments suggests that leaders motivate followers to engage consistently in reliable behaviour in the context of going ‘above and beyond’ to achieve strategic and political goals—and that this is the case across operational contexts (Baran & Scott, 2010; Fraher et al., 2017). However, like most of the other research on leader and follower behaviour that has been conducted in military settings, such work has traditionally focused on individual differences in effective leadership, rather than on group and social processes (Wong et al., 2003). Social identity theorists have challenged this emphasis, and instead argued that it is the mobilization of followers that is the ultimate indicator of effective leadership (Epitropaki et al., 2017; Subašić et al., 2011).

More specifically, social identity researchers argue that in order to mobilize followers to pursue goals, leaders need (a) to be *identity prototypes* who model appropriate behaviour (Steffens et al., 2014, 2021), as well as (b) *identity champions* who advance the group towards relevant goals (Steffens et al., 2014). Furthermore, leaders need to act (c) as *identity entrepreneurs* (Reicher et al., 2005) who not only build a strong sense of shared social identity in the groups they lead but also shape social identity content so that it aligns with higher order organizational goals of high reliability (Haslam et al., 2022). They also (d) need to work as *identity impresarios* to create structures and practices (e.g. events, rituals and habits) that embed high reliability in group members' lived experience (Haslam et al., 2020).

Although there is plenty of evidence of the importance of these different facets of identity leadership in a wide range of applied contexts (e.g. Fransen et al., 2022; Steffens et al., 2014; Stevens et al., 2020; van Dick et al., 2018), these ideas are largely untested in HROs. Accordingly, researchers have pointed to the pressing need for focused work of this form (Haslam et al., 2022; Martínez‐Córcoles, 2018). In particular, they have called for researchers to flesh out these ideas by discovering the form that high‐reliability (identity) leadership takes and seeing how precisely it engenders requisite forms of followership.

### High reliability followership (HRF)

Mention of followership draws attention to the fact that in the HRO literature (as in the leadership literature more generally; Bennis, 1999; Hollander, 1992) followers have traditionally been relegated to the margins. As a result, they are typically positioned either as passive recipients of others' leadership or—at best—as moderators of leaders' influence and behaviour (Oc & Bashshur, 2013). However, as Uhl‐Bien and colleagues point out—‘leadership cannot be fully understood without considering the role of followers in the leadership process’ (Uhl‐Bien et al., 2014, p. 89). Followership theory thus emphasizes the active role that followers play in creatively shaping, and ultimately determining, leadership outcomes (Tee et al., 2013). Here too, social identity theorists argue that the motivation of followers and their subsequent delivery of relevant group outcomes is heavily influenced by the creation of a shared ingroup social identity that serves to inform their collective action (Stevens et al., 2019). As Tee et al. (2013) point out, followers engage in actions which determine leadership outcomes largely on the basis of their own identification with the group (see also van Dick et al., 2006). So, if leaders are unable, or unwilling, to engage in identity leadership there is unlikely to be any engaged followership (Haslam et al., 2020; Reicher et al., 2005).

Again, though, this is an aspect of HROs on which previous research has been largely silent. Indeed, while there is quite a lot of work which (at least implicitly) addresses matters of high‐reliability leadership, to date there has, to the best of our knowledge, been no research at all on high‐reliability followership (HRF).

### The present research

The present research seeks to address this gap in the literature by using thematic analysis to explore the nature of HRF in elite military teams, investigating how it contributes to organizational success and reliability. In this context, ‘elite’ refers to military teams whose members are specially selected, highly trained and equipped to perform critical tasks in high‐stakes environments, such as fast‐jet aircrew and combat controllers. In line with the logic of the social identity approach, we explore whether the creation, reinforcement and internalization of shared group identity content that is aligned with HRO hallmarks might contribute to HRF that supports the achievement of reliable task completion (Haslam et al., 2022; Weick & Roberts, 1993). More specifically, using qualitative interviews with fast‐jet military aircrew and combat controllers, the study seeks to shed light on social identity, leadership and followership processes in elite military groups, and to see how these processes might support high reliability.

## METHOD

### Participants

All participants were (or had been during their military career) members of Air Combat Group (ACG)—the group responsible for fast‐jet combat aircraft within the Royal Australian Air Force (RAAF). Aircrew were recruited via emails sent through an organizational gatekeeper within the RAAF chain of command. All current fast‐jet aircrew serving in the RAAF were invited to participate, including those currently employed in non‐flying roles. Snowballing occurred naturally, with recipients also forwarding the invitation on to retired colleagues, whose contributions offered valuable insights into the persistence of identity content beyond formal service. Retired participants described similar HRF behaviours to active members, suggesting that a sense of deeply internalized identity content endures after active duty (Williams et al., 2018).

The participant pool included fast‐jet pilots, Weapon Systems Officers (WSOs—who are also aircrew) and combat controllers—all personnel specially selected and trained for critical roles in military air operations. Despite their distinct responsibilities, they share a common purpose in supporting high‐stakes, high‐reliability operations in both combat and peacetime contexts. Twenty‐four personnel participated (aircrew *n* = 21; combat controllers *n* = 3) with one individual asking to be interviewed twice. Participant demographics are presented in Table 2. Most participants (92%) were male, a proportion that reflects the level of male and female representation in most Western military institutions' fast‐jet squadrons. The rationale for selecting fast‐jet aircrew and combat controllers was that these cohorts, integral to Air Combat Group, frequently work together in high‐stakes environments, making them ideal for studying the operational dynamics relevant to high‐reliability organizations.

**TABLE 2:** Participant demographics and event context discussed at interview.

Baseline characteristics	*n*	%
Gender (*n* = 24)		
Female	2	8
Male	23	92
Age at time of the event		
19 years or under	0	0
20–29 years	9	36
30–39 years	7	28
40–49 years	2	8
50–59 years	1	4
60 years or over	0	0
Prefer not to say	6	24
Current age		
19 years or under	0	0
20–29 years	1	4
30–39 years	5	20
40–49 years	6	24
50–59 years	5	20
60 years or over	2	8
Prefer not to say	6	24
Rank at time of interview in relation to interviewer		
Higher rank than interviewer	11	44
Same as interviewer	4	16
Lower rank than interviewer	10	40
Work specialization		
Pilot	15	60
Weapon systems operator	7	28
Combat controller	3	12
Serving status (at time of interview)		
Serving	20	80
Retired	5	20
Event context for interview		
Peacetime	9	36
Operations	16	64

### Interviewer positioning

The military, and in particular elite military teams, can be difficult for ‘outsiders’ to study (Ben‐Ari & Levy, 2014). In this regard, the first author's prior training as a WSO in the RAAF provided a unique opportunity for data to be collected from an insider viewpoint. This is important for two reasons (Holstein & Gubrium, 2003; Modan & Shuman, 2011). First, it allowed participants to use their specialist language which provides the ‘cohesive glue’ for sensemaking (Modan & Shuman, 2011, p. 19) without limitations or explanation (Chavez, 2008; Dwyer & Buckle, 2009). Second, it established trust, openness and acceptance (e.g. Dwyer & Buckle, 2009; Tanis & Postmes, 2005), enabling the collection of rich and candid data. Indeed, an outsider—even one sympathetic to the military context—would likely struggle to establish rapport and grasp nuances of military interactions and context (Holstein & Gubrium, 2003).

Yet while insider status can facilitate deeper insights, it may also introduce subjective interpretations associated with pre‐existing knowledge and relationships with the military. To mitigate this, co‐authors who were naive to military contexts served as critical reviewers, providing impartial sensemaking and feedback on the interview protocol, transcripts and interpretations. In addition, the first author maintained a reflective journal, documenting analytic decisions and emerging insights. Regular discussions with the second author helped challenge assumptions and ensured interpretations accurately reflected participants' intended meanings.

### Procedure

This study received ethical approval both from the Departments of Defence and Veterans' Affairs Human Research Ethics Committee (276–20), and the researchers' university (2021/HE001041), and all participants provided informed consent prior to participation. In line with principles of Critical Incidents theorizing and methodology (Butterfield et al., 2005; Flanagan, 1954), an interview protocol was created using three theoretical approaches—Social Identity, Social Identity Leadership and HRO—as a starting point for question development (see Supplementary Material). Interviews were semi‐structured and involved several interconnected elements. Each interview began with an event prompt, in which participants described a specific incident from either a peacetime or operational context (one participant was keen to discuss two distinct incidents: one from their operational experience and another from their early career in a peacetime context—this participant was interviewed twice). The incident description stage was followed by reflective discussion, including a team identification rating question (‘How strongly do you identify with the team on a scale from one to ten?’). Its use was flexible, introduced when it felt appropriate during the interview. Typically, the rating question was used after participants had described the event, prompting them to reflect more deeply on their own sense of identity and its alignment with the group. Rather than being a quantitative measure, this question was intended to prompt (qualitative) reflection on their connection to the team and to enrich the understanding of their team‐related experiences. Comparative elements were also incorporated throughout the interviews, encouraging participants to compare experiences, provide examples and elaborate on key events. This layered approach facilitated a comprehensive exploration of leadership, followership and identity processes, in ways that also allowed for participants to share in‐depth, context‐specific narratives.

We emphasized that the event participants chose to describe was less important than how they interpreted and acted within the context of that event. This minimized the risk of overwhelming participants with emotions associated with their recall of particular missions while encouraging in‐depth narratives. Additionally, considering that the meaning of critical incidents can evolve over time (Chell, 2014), participants were prompted to reflect on whether their interpretation of the event had changed since it occurred. This temporal lens allowed for richer discussions about identity, leadership, and followership in ways that facilitated deeper engagement with abstract concepts that might otherwise be difficult to articulate (Keatinge, 2002).

After the first two interviews, the interview protocol was evaluated, and it was noted that several prompts led to discussions about the nature of leadership and of the individuals who were (and were not) contributing to leadership, including the respondents themselves. The interview protocol was modified to ensure that further interviews also captured this aspect of participants' self‐reported experience. The first author conducted the interviews, and these were recorded with participants' consent, transcribed, anonymized and imported into NVivo software. Each interview lasted approximately 1 h (ranging from 55 to 65 min), providing substantial qualitative data for analysis.

### Data coding and analysis

Themes were generated via thematic analysis that was informed by a critical realist research perspective (Braun & Clarke, 2024) adapting Saldaña's (2021) three‐phase approach to coding the dataset. The first phase involved initial coding and consisted of repeated and active reading of the transcripts. The first author used a mixed deductive and inductive approach, to this process such that codes were both interpretative (researcher‐generated based on preparatory work informed by social identity theory, social identity leadership theory, and HRO theorizing) and data‐derived (driven by novel and relevant features of participants' accounts, which were data‐driven rather than theory‐informed, allowing for capturing unexpected aspects of their experiences; Byrne, 2022; Fereday & Muir‐Cochrane, 2006; Saldaña, 2021). A mixed deductive and inductive approach was used. This approach combined theory‐informed coding, based on social identity, social identity leadership and HRO theorizing, with data‐driven coding that captured any novel features evident in participants' accounts. Deductive and inductive coding were conducted concurrently throughout the first phase of analysis, ensuring theoretical patterns were examined while allowing unexpected insights to surface. The entire dataset was coded to ensure comprehensive and systematic coverage, balancing theory testing and extension.

Inductive coding contributed specifically to the HRF themes by revealing overlaps, complexities and contradictions within the pre‐existing HRO principles, rather than simply mapping onto them. This interplay between the deductive and inductive codes helped to identify the nuanced ways in which participants' shared social identity content aligned with, or diverged from, HRO principles. To account for the insider perspective and to ensure richness in interpretations of meaning, a second coder was used to identify patterns in the data that the first author, as an insider, might take for granted. The first author and second coder worked independently, coming together at regular intervals to discuss and compare their observations and interpretations. This collaborative process was intended to improve the researchers' sensemaking process by reflecting openly on their distinct positioning and potential differences in their interpretation of the data.

In the second phase, the first author used the initial code list to conduct pattern coding, enabling patterns in the dataset to be identified and organized into loose themes. These themes were considered by the full research team, with each author contributing to both the merging and breaking down of the themes. The first author then read all coded extracts for each theme to ensure they followed a coherent pattern. To ensure their comparative fit, the themes were reviewed by the research team to ensure intra‐theme homogeneity and inter‐theme heterogeneity (Clarke & Braun, 2017).

The third phase involved analysing the data to identify structure in, and connections between, the themes. First, the research team worked together to refine and define each theme. Second, the first author organized the themes into a coherent narrative which fitted into the broader, overall story of the data. Following this, the research team reviewed the coherence and organization of the themes in the process of final drafting. All quotations selected for inclusion in the final paper underwent a validation process, in which the respective participant was contacted to check that the transcripts accurately reflected their original statements. Participants were also asked if they were comfortable with the use of the quotation, and to check the level of disclosure to ensure that their anonymity was preserved. To further protect their identity, participants were identified only by their role (e.g. Pilot, WSO, Combat Controller). This was because—given the small population from which they were drawn—linking quotes to individuals' participant numbers or characteristics (e.g. gender, age, active/retired status or specified missions) would have the potential to compromise anonymity. This approach ensured participants felt comfortable sharing sensitive information openly while also preserving data integrity. Participants' responses were often concise, reflecting the structured nature of their professional communication and the environments they operate in. Given this, many extracts presented in the manuscript are necessarily brief. However, longer extracts providing richer context are available in the supplementary materials.

## RESULTS

Data analysis revealed three themes: (a) the strength and alignment of shared aircrew identity, (b) the role of identity leadership in crafting and maintaining that shared identity (and promoting followership) and (c) followership reflecting reliability‐reinforcing shared identity content. In addition, within the third theme, five subthemes reflecting the behavioural aspects of HRF were identified, including specific dimensions of shared identity content.

### Theme 1: The strength and alignment of shared aircrew identity

Data indicated that all participants felt a strong sense of identity (as a pilot, WSO, aircrew or combat controller). While participants reflected on specific events when prompted, much of their discussion centred on a broader, persistent sense of identity. This tendency appeared to be linked to their desire for clarity and a lack of ambiguity when discussing team dynamics and shared identity content, even when describing shifting group boundaries. The thread of a shared group identity ran through all participants' narratives and was exemplified by the statement: ‘Being Growler aircrew is the central component of my professional identity and to an extent my professional identity is a significant component of my personal identity’ [WSO].

Participants also reported levels of identification were stronger within their group when compared to those outside the group and their perceived strength of this shared identity was also very high—with 91% of participants rating their identity strength as 9 or 10 on the 10‐point scale, and 23% rating it above 10 (i.e. off the scale). One pilot reflected: ‘back when we are talking XO [executive officer] of the squadron, I was a ten without a shadow of a doubt, in fact I was about a 15’.

This reflects not only the strength but also the centrality of their aircrew identity within their broader social network, where their professional identity forms a core component of their personal and group memberships, consistent with literature on identity centrality in military contexts (Haslam et al., 2020). This reported shared identity was also clearly aligned with participants' confidence that others in the group held the same beliefs: ‘You're confident in the leadership that you have and you're confident in your peers that are in the same group’ [Pilot].

The participant who was interviewed twice reflected on incidents from both peacetime and operational contexts. Across both narratives, this person described similar experiences associated with similar shared aircrew identities focused on reliability, teamwork and operational excellence. Although we did not specifically seek to explore differences or similarities between these two contexts, this consistency, along with consistency among other participants (including those who had retired and who were still serving), suggests that these identities are not especially context‐dependent but are instead embedded within the group's shared identity and shape actions across diverse environments and time.

However, perceived alignment was not limited to personnel within their immediate aircrew or combat controller group. Instead, personnel saw alignment as extending to others when it was necessary to form a temporary work group for specific tasks. That is, the shared identity could expand or contract to fit those personnel and groups who participants judged as sharing their operational goals in a given context. In several instances, participants also expanded the group boundary to include other military groups who they believed shared compatible values. Some aircrew participants discussed events where they were not in flying roles, but instead were working in teams pulled together from different military specializations, noting that in these contexts they applied their shared aircrew identity content (in the form of values and behaviours) to the newly formed group of disparate personnel. This enabled the participants to create and embed a sense of ‘us’ for the newly formed group in a way that supported integration and alignment. As one pilot put it: ‘INTELOs [Intelligence Officers] and maintenance people operate very differently when they are in a fighter squadron, and they will tell you that they take on the personality and the culture’. [Pilot].

Yet as well as expanding the boundary of their ingroup when this made operational sense, participants sometimes also contracted it. In particular, they did this by emphasizing the firm boundaries which separated the group's shared identity from groups (or individuals) who were not seen to represent the values or behaviours of the group. For example, one responder noted that their team had become ‘super tight‐knit and really cohesive … because we all kind of banded together against this common enemy of the exec [executive]’ [WSO]. Some participants also created significant boundaries around the group, thereby separating the group and themselves from the wider organization (e.g. noting ‘I feel quite apart from the rest of the military outside of the fighter force’ [Pilot]).

### Theme 2: The role of identity leadership in building shared identity

Although participants often made passing reference to rank or hierarchical status, they typically zeroed in on behavioural characteristics of the leader in ways that indicated these were more influential for followership than the person's rank or status in the organizational hierarchy. For example, one WSO observed ‘the CO [Commanding Officer] was a charismatic leader and very good at motivating people to work for him’. Several participants also noted the importance of informal leaders. These informal leaders were also seen to embed shared identity content through their influence ‘in setting the environment, the standard, the approach, the maturity towards it all’ [Pilot].

Analysis revealed that three factors played a significant part in whether a ‘leader’ evoked followership within their teams. These mapped broadly onto three of the four components of identity leadership—(a) consistently displaying the core behaviours valued by the group (identity prototypicality), (b) group goals over personal interests or glory (identity advancement) and (c) actively contributing to the creation of a highly reliable team (identity entrepreneurship).

Being one of ‘us’ (identity prototypicality) was universally valued by participants, and centred on a person's credibility as a group member, both in terms of technical skill and an individual's experience and recency. Moreover, these aspects of prototypicality—technical skill, experience and recency of flying—all had clear links to highly reliable behaviours. To establish credibility, participants referenced expert roles held within the group. Indeed, a third of aircrew participants listed either a Fighter Combat Instructor as the most influential leader who evoked followship within their immediate team, or individuals holding the highest level of flying category (e.g. ‘He's always someone from the very beginning that I've always looked up to … he is an A‐Cat [A‐Category]’ [WSO]).

A further aspect of prototypicality was the value ascribed to the leader's perceived *conformity* to shared identity content—also reflected in the problems that were seen to arise in the team when individuals acted in idiosyncratic ways. In particular, when team members went against the group's shared identity content, and thereby challenged the group's identity strength and alignment, respondents indicated that they were ‘re‐educated’ by the team to bring them in line with the group's expectations. This is reflected in the observation of one combat controller that ‘if that trend continued you would probably look at some sort of social distancing and that horrible trajectory, where people realise [that] they are no longer part of the capability’.

Also notable in this regard was the fact that there were many instances where hierarchically appointed leaders were seen to be unable to exert influence and lead effectively because they misunderstood or misread the content of the group's shared identity content and therefore were not seen as prototypical. This is seen in the observation of one pilot that:

nobody wanted to be like him [the CO], because he was mad, we said he was mad; [we didn't want to …] live in a tent, drink insane amounts of beer, work insane long hours, party for the other hours of the day, and sometimes sleep when you had to.

This resulted in some officers (commissioned and non‐commissioned), who were notionally leaders, not being able to do much actual leadership; when talking about their CO one WSO noted that ‘day‐to‐day, they didn't have much influence’.

Second, it was not enough for a leader to be prototypical and exceptionally good at their job for them to inspire followership. Advancing group goals—‘doing it for us’—was also necessary. When participants came to the view that a leader was failing to advance the group, this generally reflected a sense that they were putting their *own* success above that of the group. For example, in explaining why they were unenthusiastic about their CO, one pilot described him as being ‘a very, very, very, very capable fighter pilot, but God you didn't take your eyes off him, because he'd leave you behind’. Relatedly, respondents also took a dim view of leaders who were perceived to be self‐interested (e.g. ‘nobody trusted him to not look out for himself and sort of go for glory’ [Pilot]). Participants also explained how this type of behaviour had a negative impact on the group (e.g. ‘the outcome for the unit was a lack of faith in the command at that point… and mistrust’ [WSO]). And, again, this meant that although those who were seen to behave in this way may have had the designation of ‘leader’, they were nevertheless rejected by the group—and therefore unable to actually do leadership. As one WSO observed of his CO: ‘We didn't identify with him. I guess the only group cohesion is that we weren't with him, I suppose we were against him. … Screw the boss!’

Finally, participants linked effective leadership to a person's ability to give the group a shared sense of focus and purpose via forms of identity entrepreneurship that cultivated a sense of ‘us’. In the words of one pilot, ‘a lot of the time it's about aligning people's personal goals with the overall goal and seeing how they get there’. In many cases, this identity entrepreneurship was observed in those who were formal leaders. However, this entrepreneurial aspect of identity leadership was also observed to be something that was distributed across the group: ‘you get shaped a little bit by the community and that happens because the community rewards the sort of behaviours that you want to see in people’. [Pilot] Indeed, as another pilot noted, shared group identity content was often already embedded in the team so that all the positional leader needed to do was to go with the collective grain: ‘his job was probably quite easy in that way. We joined the Air Force to fly, and fight … so all of us wanted to do it, so I don't think he had to try very hard’.

### Theme 3: Followership reflecting reliability‐reinforcing shared identity content

In addition to the two themes identified above, thematic analysis identified a third theme that pointed more specifically to ways in which shared social identity content related to, and formed a basis for, HRF. This theme lays the foundation for a deeper exploration of HRF, which is further examined in five subthemes. These subthemes illustrate followership‐related responses that align closely with the five principles of HRO identified by Weick and Sutcliffe (2015); see Table 3.

**TABLE 3:** Hallmark practices of HROs and HRF.

HRO principles	High‐reliability followership		
	Practice	Entails	Looks like
Reluctance to simplify	Engaging with ambiguity	Understanding that ambiguity and uncertainty are likely to be present, and taking steps to actively manage inevitable uncertainty and ambiguity so as to minimize the possibility of even minor adverse events	We know that ambiguity and uncertainty are inevitable in our work. Therefore, we seek out uncertainties and take active steps to manage them. We do this, even though we know that we would not have all the solutions. We actively make our behaviours predictable when we are faced with unexpected or ambiguous events
Pre‐occupation with failure	Attending to failure	Being motivated to anticipate and respond to problems as a core part of one's work	We feel a strong sense of responsibility to improve, are motivated to root out causes of errors and are honest when appraising our own roles in anticipating or responding to problems
Commitment to resilience	Perseverance through setbacks	Persistently working through any problems that arise and bouncing back in the face of setbacks	We do not wait for mistakes to happen, and we persistently evaluate what we do to ensure that we are employing best practices in our daily work. When things go wrong, we are able to quickly regroup and carry on. We support each even if actions led to task failure but only if those actions were consistent with our group identity
Deference to expertise	Proffering expertise	Make one's expert contribution proactively when it is needed	We know that our expert contribution is valued, sometimes more than organizational hierarchy. We actively maintain our expertise and will provide it when required for a task. We also know the limitations of our expertise and rely on credible subject matter experts when required
Sensitivity to operations	Striving for team success	Understanding and appropriately addressing matters that are critical for task success	We understand that we contribute a critical component of overall team success, and we internalize that purpose. We are clear in our task focus and act to ensure there is excellence in all that we do

Critically, the followership‐related responses described in Theme 3 were directly shaped by the identity leadership processes outlined in Theme 2. More specifically, leaders' efforts to craft, embed and reinforce shared identity content provided the behavioural expectations that guided how participants enacted followership in ways that suggested identity leadership was a driving force behind followership behaviours that reinforced high reliability.

**FIGURE 1:**
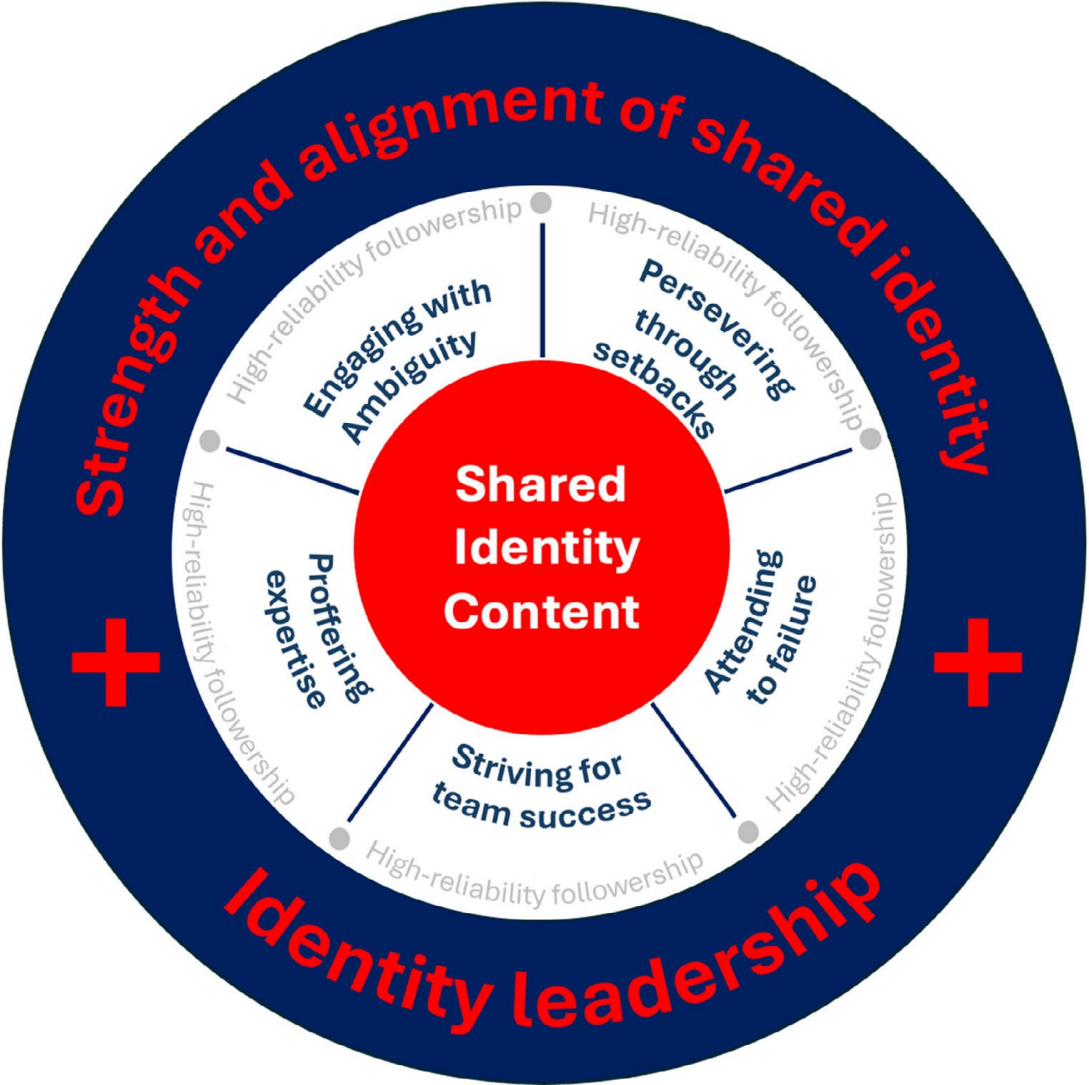
Model of high reliability followership.

As Figure 1 indicates, each of these five subthemes relates to different aspects of HRF. These grounded the group's shared social identity content in practical behaviours that support high reliability. Critically, these behaviours were seen by the participants as normal and expected group behaviours—constituting what ‘we’ do, and how ‘we’ behave. In what follows, each of the five themes and the corresponding practical behaviours are discussed in turn.

#### HRF subtheme 1: Engaging with ambiguity

Engaging with ambiguity represents the group's followership response to the HRO principle, *reluctance to simplify*. This involves the team recognizing the VUCA‐context in which it operates and responding practically to complexity through proactive engagement with ambiguity and uncertainty. The team also understands that simplifying organizational activities, or the context that the team is working in, might impede the group's ability to complete the task. Accordingly, instead of simplifying, team members reported engaging with ambiguity through a set of shared—and expected—followership behaviours. Leadership efforts to embed shared identity content provided the cognitive and behavioural frameworks that enabled team members to proactively engage with ambiguity, rather than attempting to eliminate it altogether. More generally, they articulated a shared understanding that uncertainty and ambiguity are an inherent part of their group's lived experience. As one pilot explained: ‘there is going to be risk in any fight we are going to, you can't know exactly what's going to happen’. As the comments of another pilot indicate, this understanding was also seen to be bound up with the content of the team's shared identity: ‘You've got to make sure that you've taken that ambiguity and you've brought it down to a mission set that everyone understands how they are going to operate’.

As part of this shared identity, content participants acknowledged a responsibility to manage uncertainty and ambiguity, as far as reasonably practicable, so as to eliminate the possibility of even minor adverse events. Furthermore, engaging with ambiguity was generally seen by participants as a necessary process that allowed them to perform better. This is captured in the reflections of one pilot that ‘It [the military operation they were leading] was uncertain, but to be honest with you, I always like it when things fall apart because then you can paint outside the lines fundamentally to get the job done’.

Participants also expressed a shared understanding and acceptance of the inescapability of VUCA situations, recognizing the inevitability of uncertainty. This is exemplified by one pilot's reflection that ‘I think there is always uncertainty in those missions, but I don't think that's something that impacts your thought process or your game plan, the uncertainty is certain’. The team actively attempt to reduce ambiguity as much as possible, so as to increase the likelihood of task completion. If the team are unable to reduce ambiguity and uncertainty, they modify their actions to ensure the team is able to work within the VUCA environment in ways that support task completion. Participants were deliberate in their observations on how they sought to reduce ambiguity through both proactive planning and reactive behaviour, and this was central to their overall strategy for managing the complexity of their operational environment. Here, team members reported engaging proactively with Standard Operating Procedures (SOPs) to ensure that they helped them engage with VUCA. But, noting that in VUCA environments, there is often no time to discuss a plan of action, team members also reported reflecting on shared identity content to reactively gauge what constitutes ‘acceptable’ behaviour in response to ambiguity. In this way, each team member had an expectation of how group members—including themselves—should, and was likely to, respond when faced with ambiguity. This serves to define ‘acceptable’ behaviour in response to ambiguity. In particular, if the situation is highly ambiguous, respondents noted that the group would often become more predictable and take less risk to eliminate the possibility of adverse events. As one put it ‘I think we use complexity as a throttle to control our risk appetite’. [Pilot].

Finally, team members had a sense that recognizing and reducing ambiguity can only be accomplished through a commitment to continuous training specifically for VUCA contexts. However, because of the inherent uncertainty of their operations, it is also accepted that what the team has trained for might never eventuate or that what does eventuate might be fundamentally different to what the team has trained for. Thus ‘most of what we do as aircrew, is prepare ourselves for the worst‐case scenario’ [Pilot].

#### HRF subtheme 2: Attending to failure

The HRO principle, *preoccupation with failure*, describes how an organization must continuously attend to anomalies and minor adverse events, as these could be harbingers of larger problems (Weick & Sutcliffe, 2015). As a corollary of this principle, it was clear that the group internalized *attending to failure* as a necessary part of HRF and a key component of its shared identity content. In this way, participants reported that they were motivated to anticipate and respond to problems as a core part of their work: ‘We were all primed… I guess that was a trigger where we were like, yep, okay. … Recalibrate’. [WSO].

Once an issue is recognized, there is a team expectation that personnel will respond to minimize the chance of failure: ‘It's just inherent in what we do. … I guess in our career we are encouraged to actually identify reasons for errors, mistakes, successes and we take it down to a thing that can be done again’ [Pilot]. As a part of this process, personnel whom the team believe are attending to failure are rewarded, even when failure actually occurs. In particular, reward takes the form of group acceptance. Thus, one pilot reflected on the treatment meted out to a fellow pilot whose actions had led to an incident during training. The pilot who caused the incident took responsibility for his failure and demonstrated a proactive attitude towards improvement, thereby receiving the ‘forgiveness’ of the group: ‘We all went to the bar and he bought a carton of beer and we all drank his beer and everyone was happy after that, but at the time, the day of the incident, he was mortified’ [Pilot].

Participants indicated that attending to failure was embedded in the team's identity, with a shared responsibility to improve, conduct honest self‐appraisals and engage in motivated fact finding when things go wrong. Here, participants acknowledged that there was always something that could be improved, also stating it was a team expectation that group members displayed a commitment to actively making improvements. Respondents noted however, that this sense of responsibility to improve was only beneficial if team members were able to engage in honest self‐appraisal. These followership behaviours are a direct reflection of leaders modelling and championing values of openness, accountability, and integrity, which then become internalized by team members. They also noted that this was essential for the effectiveness of the group as a whole. Thus, one pilot reflected that ‘there's a general desire and a general expectation of complete integrity and complete openness and honesty that if you don't have, you will eventually fail in the fast‐jet community’. This level of self‐appraisal relies on team members feeling psychologically safe enough to reflect on their actions without fear of negative repercussions:

In the debrief it was obviously my fault and it was…I found it not hard to admit. It's disappointing, but I had no sort of compunction with going hey, that was …that was my bad. Standing up in front of the squadron talking about the assumptions I made and etc like that. [Pilot]

Group members also reflected on the importance of motivated fact finding to get to the issues at the heart of the failure. This was seen when one pilot reflected on an incident on operations: ‘We just went through the process of trying to have a look, okay, how do we stop this happening again or what else could we have done?’ However, there was a recognition that this fact finding only supports reliability when it stimulates both team and individual improvement. Participants observed that there are two reasons why fact finding allows the team to move forward. First, it educates the team in the details of failures in ways that enable its members to recognize ‘weak signals’ or hazards which, if addressed, could prevent or reduce the likelihood of an event happening in the future (Weick & Sutcliffe, 2015). Second, the business of fact finding itself generally leads to processes or procedures being refined or developed so as to reduce the likelihood of reoccurrence. Thus, in the wake of an incident in a fast‐jet exercise, one pilot remarked ‘We looked at all different levels of procedures and the crewing and so forth through to the commanding’.

#### HRF subtheme 3: Persevering through setbacks

Participants noted that team members were expected to be tenacious in continuing with the task in hand, even in the face of setbacks that arose from either ambiguity or failures. Indeed, this was seen to be another core aspect of their shared social identity. This subtheme can be seen as an HRF action response to the HRO principle of *commitment to resilience*. At an organizational level, commitment to resilience ensures that the organization is not disabled by failure and is able to recover from failures quickly (Haslam et al., 2022; Weick & Sutcliffe, 2015). On the ground, though, it is the team's *persevering through setbacks* that means that when it experiences setbacks, it is able to stick the course.

Perseverance through setbacks was evident in participants' descriptions of their team's ability to compartmentalize and rationalize, which was described as a key behaviour expected of all team members. For example, as one pilot noted, if one aspect of a mission does not go according to plan, team members are able to recognize, acknowledge and actively prevent the mistake or failure from derailing the rest of the mission: ‘Once we sort of, had finished what was required in that area, we just tracked away to go and do what was next for us and started off‐loading that information back’. [Pilot]. This ‘in the moment’ team behaviour to take stock and move on does not mean that any failures will not be re‐visited at a later date. Indeed, failures and mistakes are thoroughly addressed through the behaviours associated with the previous subtheme *attending to failure*. However, it is not only failures that require resolution. As an aspect of the current subtheme, participants described how their team was committed to continually evaluating *all* work practices, as this was understood by the team to be an essential aspect of its ability to achieve reliable task performance. This evaluation of work practices is achieved through the team's continual and dynamic debriefing. The continual debriefing of all aspects of work practices, not only failures, produces knowledge and understanding, which subsequently become part of the group's shared knowledge that supports high reliability. As one pilot remarked, ‘You can absolutely go to town and hash it out as much as you want in the debrief and it's only ever going to get better from there’. This debriefing process is not only continual, but also dynamic, reflecting the team's inherent understanding that because of the complexity of the technology they use and the VUCA‐ness of the environments they work in there is always room for improvement. In this vein one WSO reflected that: ‘If you find the mission was all good and there's no downside, there is a downside, you just haven't been able to identify it’.

Accordingly, when things do not go to plan or decisions are made which limit the team's ability to complete the task, team members need to be able to work through this and find the positives alongside the failures. Team members' ability to compartmentalize and persist was reinforced by leadership practices that instilled confidence in the group's shared ability to recover from setbacks, ensuring alignment with organizational goals. This allows them to regroup and persevere as a team. Even when a decision had resulted in task failure, the ability to reflect on their actions allowed group members to conclude that they had still made the ‘right’ decision based on the group's identity content. Thus, after a tactical breakdown in communication, one pilot reflected ‘I think we all agreed in the end it was the right call, although we upset the JTAC [Joint Tactical Attack Controller] and it did result in us ultimately letting these guys get away’.

#### HRF subtheme 4: Proffering expertise

The HRO principle of *deference to expertise* highlights the need for an organization to recognize and draw upon relevant expertise whenever it is needed. This ensures that those who are most qualified to provide input are empowered to do so regardless of their position in an organizational hierarchy (Weick & Sutcliffe, 2015). The HRF counterpart to this is a response that sees *proffering expertise* as another important aspect of social identity content. Here, team members have a shared belief in the value and normativeness of any expert contribution they might make and also believe they will be safe and supported when making it.

First, participants emphasized that whilst being able to proffer expertise requires team members to have knowledge and skill in a specific domain, participants also remarked that knowing their own limitations was critical:

He's exceptional at what he does, always trying to be better, … even he would admit to coming to someone like me if I had more experience in a certain field to ask questions and stuff and would actively do that with people. [WSO]

In particular, they pointed to the fact that no individual or team contains all the knowledge or answers to every question. As one pilot put it ‘A P3 driver would never aim to talk about air combat tactics any more than a fighter jock would tell you how to do ASW [anti‐surface warfare]’. Here the strength of the organization lies in the fact that each team, and each of the members within it, has ownership of some part of the organization's knowledge and this is a basis for a shared understanding that it is necessary and normative for personnel to proffer their expertise. As explained by one pilot: ‘Air Force is about a knowledge economy. So, it is about SME [subject matter expert] input being the most valuable contribution, above rank, above qualification, above all of the other social structures that we put around it’.

Proffered expertise, though, needs to be both useful and accurate. If it is not, it undermines the credibility of the individuals and teams who provide it. Accordingly, personnel need to know the limitations of their experience and knowledge so as not to over‐promise on their capacity to complete tasks. An inherent part of this is the team's awareness and acceptance that the context in which the organization operates is too complex for any individual or team to know or control. As one pilot put it:

We respect specialisation above rank, and I think that comes back to the basics of even maintaining an aeroplane. If the corporal tells you the aeroplane is not serviceable to fly, you're not going to argue with them, are you? That's his specialty.

The second aspect of proffering expertise concerns the importance of team members maintaining credibility. Credibility reflects the fact that a person's expertise is valued and trusted by the team. Participants indicated that credibility is achieved and maintained through a constellation of visible actions, which all team members—including the CO of a squadron—must actively engage in to maintain their group credibility.

Finally, although military life is hierarchical, team members universally acknowledged that, on its own, rank is not an indicator of expertise in so far as they valued experience over hierarchy. As one of the pilots said of his former CO, ‘It was never a, I'll knock on his door and stand to attention because he's a wing commander, and I would absolutely pull him up when he screwed up and he would expect me to as well’ [Pilot]. Also, in line with this principle, another pilot reflected that ‘I've seen some very experienced older dudes come back on refresher conversion and be super humble and listen to the fresh‐faced C(MQ), because at that moment, he's the most proficient’.

#### HRF subtheme 5: Striving for team success

*Striving for team success* was the group's follower response to the HRO principle of *sensitivity to operations* which relates to the organization's awareness of what is going on within the interactions, activities and processes that constitute organizational systems ‘on the ground’ (Weick & Sutcliffe, 2015). In response to this, participants noted that it is expected that group members share both a commitment to understanding, and a responsibility to appropriately address, matters that are critical for task success. As a result of this, in the words of one WSO, leaders have ‘the ability to give someone an intent without having to describe exactly what you need’. Analysis indicated that this commitment and responsibility on the part of front‐line personnel underpins key mindsets that its members share. In particular, participants indicated that they understood the seriousness and potential ramifications of not achieving task success because, in the words of one pilot:

Making a mistake or misjudging a risk or putting the wrong decision [sic] is potentially going to put someone in a bad situation where they are risking life and limb, or someone else is going to have to risk life and limb to go and get them.

This understanding also extended to an awareness that group actions form part of a larger strategic context in which the group's actions had consequences for achievement of the overall strategic goal. Accordingly, participants reported having a responsibility to ensure that, however small their contribution is, their actions are appropriate and aid the overall strategic goal. In the words of one pilot: ‘You are never going to win the war with the next bomb, but you sure could lose it’.

Respondents again discussed the practical ways in which the team lived out their commitment to team success. First, they noted that all team members need to share a common task goal such that they are focused on the same outcome and have clear task focus. However, beyond a clear focus, team members also need to have a practical understanding of their role in securing key outcomes. This involves owning the purpose such that, in the words of one WSO, ‘I can't just kick the can down the road and make it someone else's problem’. Participants also recognized that due to the nature of the tasks they perform and the contexts in which they operate they might only ever have one opportunity to complete their task. For this reason, they need to be committed to excellence, so that they perform those actions as well as they possibly can. Again, this commitment was identified as a shared value of the group and one that was central to the way in which aircrew understood their group membership. As one pilot succinctly put it ‘Whatever your job is, we are all about doing that thing super well’.

### Counterfactual evidence

As the interview protocol primarily focused on leadership and group interaction during events, responses naturally centred around behaviour in the context of group interactions. Therefore, the results presented above should not be interpreted as implying a consistent presence of HRF or its ability to explain all behaviours. While our findings provide a possible framework for understanding the behavioural manifestations of high reliability among team members, we also searched the primary data for any counterfactual evidence or data that conflicts with the notions we have described above in order to test our assumptions and to counter confirmation bias (McSweeney, 2021). We found that, in 40% of the interviews, participants shared instances of behaviour exhibited by group members or outsiders that was counter to the practices of HRF. These behaviours, however, were not regarded positively. Instead, participants used such behaviour, which they perceived to be unacceptable, to either provide an explanation for an adverse event or task failure, or to justify the exclusion of individuals from the group (e.g. ‘The outcome for the unit was a lack of faith in the command at that point—and mistrust’ [WSO]). It is worth noting that participants often positioned counter‐HRF behaviour as a cause for the breakdown of the team's ability to complete operational tasking (e.g. ‘It's literally as a result of two senior guys on the formation, one who's the CO, pushing the rope…and eventually the safety happens’ [Pilot]).

Another area that stood out was the reported strength of identification with the group by participants. We have argued that shared identity is central to HRF, and therefore our attention was drawn to commentary of the 9% of participants (*n* = 2) who rated the identification with their group as 8 or lower. Here individuals assigned different ratings to different aspects of their careers. For example, one participant identified as a five or six when considering their role as an aircrew instructor in a training squadron. However, this was juxtaposed with their strong sense of identity as an aircrew member in a frontline squadron, describing it as an integral part of themselves. Relatedly, another pilot observed that their identification reached its lowest point when they perceived themselves as lacking credibility. This link between credibility and identification has clear links back to proffering expertise, suggesting that the relationship between social identity and HRF is more circular than linear. Similarly, a combat controller—who rated themselves as a nine—stated, ‘I never rate something a ten’, going on to emphasize the unpredictable nature of their work and the need to be prepared for unexpected challenges; while a WSO reflected that, ‘I wouldn't say it's a ten because that leaves no room for growth’. These responses suggest that even those few respondents who did not rate their identification as being extremely high may nevertheless have deep alignment with their group and the principles of HRF. In particular, their commitment to perseverance, along with the other key aspects of HRF, appeared to permeate all facets of their interactions and to have an enduring impact on their mindset and approach.

## DISCUSSION

The goal of the present research was to explore the nature and experience of high reliability from the perspective of front‐line teams. In contrast to previous research which has focused (implicitly) on *leadership*, our focus was on *high‐reliability followership* (HRF)—the process through which goals of high reliability are willingly and actively pursued by those at the organizational front line. The impetus for this study stemmed from the pervasive leader‐centric approach often found in research into HROs (including the military), and the descriptive focus of HRO theorizing (Haslam et al., 2022; Wong et al., 2003). Responding to the call by Haslam et al. (2022) for investigation of the forms of followership that high reliability (identity) engenders, and that help to sustain it, our research points—for the first time—to ways in which social identity processes are central to the creation and maintenance of HROs. More specifically, our findings are (to our knowledge) the first to clarify ways in which identity leadership helps to build identity strength, content and alignment within organizational teams in ways that are then translated into engaged followership on the part of those responsible for ‘doing’ high reliability.

A central thread running through all participants' responses was the strength and alignment of shared aircrew identity built around high‐reliability identity content. Indeed, identity strength and content were, at times, invoked strategically so that group members could achieve broader goals. For example, when necessary, respondents adjusted their understanding of shared identity to encompass personnel from other groups whose knowledge, skills or abilities were essential for task success. They were also sensitive to the ways that this shared identity was crafted and maintained by identity leadership. Importantly though, this leadership was not necessarily aligned with, or the consequence of, a person's hierarchical position. Instead, leaders (i.e. those capable of exerting positive influence over the group and its actions) were those individuals who were seen to best represent the core behaviours valued by the group, who advanced group goals and who actively contributed to the creation of the team (along lines suggested by Haslam et al., 2020; Steffens et al., 2014). This reflects the fact that effective leadership was perceived to centre on identity advancement and identity entrepreneurship—not just identity prototypicality. In this way, the core behaviours valued by respondents were shaped by the group's shared identity, tailored to the specific context of the group's reality, and held together by and through identity leadership. It is this combination of engaged followership and core behaviours that are internalized as definitive of shared group identity that thus forms the basis of HRF. In fact, the consistent enactment of shared identity content across peacetime and operational contexts underscores the stability of HRF. Regardless of mission demands, participants described a coherent approach to teamwork and operational reliability, suggesting that shared identity processes provide a durable foundation for high‐reliability behaviours (Haslam et al., 2020; Weick & Sutcliffe, 2015).

As well as finding social identity and the leadership that builds it to be important, we also found evidence of the relevance of the five principles outlined by Weick and Sutcliffe (2015). However, these principles took on a somewhat different form when looked at from the perspective of followers. Moreover, although each HRO principle had a closely aligned follower‐related response, there was not a direct translation of the principles of leadership into the behaviours and responses of followers (Uhl‐Bien et al., 2014). As a result, the nuances and practicalities of team members' behaviour could not be directly inferred from Weick and Sutcliffe's principles (Weick & Sutcliffe, 2015). Importantly too, each of the HRF hallmarks could be broken down into action‐responses. These served as a foundation of the group's shared social identity content that represented the practical behaviours of the group that support high reliability—who ‘we’ are, what ‘we’ do and how ‘we’ behave.

These action responses to the principles of HRO were also not mechanical, obvious or repetitive. Instead, they were graded, textured and creative—so that the shared social identity of the group and its instantiation in HRF practices was always adaptive and dynamic. What stood out here was also the accessibility of HRF for the participants. In particular, it was evident that they had a clear capacity to articulate the specific behaviours that contributed to making the group highly reliable in a specific operational context. This was coupled with a deep sense of responsibility that they reported feeling towards the team, and by extension, to the wider organization. This responsibility was not superficial, but rather internalized as a personal responsibility which manifested in the proactive and active engagement of followers in pursuit of high reliability. There was thus an intricate interplay between followers' practical responses to each HRO principle and their internalizing of these responses as an aspect of their shared social identity.

In this way, it is apparent that the effectiveness of HRF hinges on the shared identity content held in the collective mind of the group, and the leadership that moulds it. In these terms, the textured layering of HRF can be seen as the outcome of followers heedfully interrelating and working together in ways that are informed by a collective mind (along lines described by Weick & Roberts, 1993). It is therefore the combination of engaged followership and an internalized set of shared highly reliable behaviours, as cultivated by identity leadership, that allows the group to achieve near‐perfect task completion.

This study contributes to existing HRO theorizing by identifying HRF as a core aspect of the behaviour of teams in HROs. It provides initial evidence that HRF is comprised of distinct sets of behaviours that are developed by the group and internalized by its members as a shared identity content in ways that allow them to live out the principles of HRO. Although this study focuses on the identification of HRF within military teams, there are parallels with other groups in complex organizations in which group members are required to consistently behave with high reliability and where contexts are VUCA. Accordingly, as was the case with the foundational research of Weick and Roberts that was conducted on aircraft carriers (1993), the insights derived from the present research clearly have the potential to be applied well beyond the military settings that were our focus here. Moreover, this study fleshes out and extends previous social identity theorizing around leadership (e.g. Haslam et al., 2020) by linking social identity processes to HROs. In particular, our findings show how shared social identity within teams can be aligned with reliability‐reinforcing identity content, providing a deeper understanding of the ways in which social identity shapes the behaviours that are crucial for high reliability in complex organizations.

### Limitations and future research

As with all research, this study had a number of limitations which it will be important for future research to address. First, although the data coding in this study was conducted using a mixed inductive and deductive approach, the codes were heavily reliant on previous HRO theorizing for code generation. Specifically, the coding process was informed by the established concepts of the collective mind and the five HRO hallmarks. There are questions here, though, about how distinct these hallmarks are from each other and how they relate to other concepts in the organizational literature (e.g. safety climate and resilience engineering, see Casey et al., 2017, Griffin et al., 2016, Hollnagel 2006). Accordingly, there would be value in future research using quantitative methods to examine heterogeneity in the hallmarks and to calibrate their conceptual distinctions and measurement properties. Such investigations would help to refine the conceptualization and operationalization of HRO hallmarks and HRF practices, in ways that would increase confidence in our theoretical and practical claims.

Second, while the first author's military background facilitated trust and open discussion, collaborative analysis helped ensure that interpretations were grounded in the data rather than shaped solely by prior knowledge. However, this background may have also influenced the nature of participant responses, as familiarity with key operational concepts likely reduced the need for participants to elaborate on well‐understood themes, contributing to the brevity of some extracts. While this facilitated efficiency in interviews, it may have also led to assumed shared understandings. This is mitigated by the inclusion of longer extracts in the supplementary materials, ensuring transparency in the analysis. An interesting avenue for further research would involve exploring how elite groups respond to interviews conducted by outsiders and whether this qualifies the conclusions of the present analysis in any way.

Additionally, while participants consistently described HRF behaviours across peacetime and operational contexts, contextual differences such as mission complexity and team composition may still influence how these behaviours are enacted. The observed consistency suggests a stable foundation in shared identity content, but future research might explore these dynamics further, particularly in contexts with more diverse operational demands.

Our study's participants were able to provide data related to HRF in the military context. Accordingly, as we noted above, to broaden our understanding of HRF, it will be important for future research to examine HRF in other HROs—particularly those with different norms, values and cultures. Additionally, while our primary focus is on HRF, our findings suggest that there is an interplay between formal and informal leadership within these high‐reliability environments. Understanding how informal leaders can emerge and influence followership that maintains reliability, especially when formal leaders fail to demonstrate identity leadership, is an area ripe for further exploration. Future studies could further delineate the roles of formal and informal leadership in HRO settings to see how they impact team dynamics and high‐reliability behaviours.

Moreover, building on this work, developing a valid and reliable scale of HRF would be a useful way of quantitatively testing the impact of HRF on meaningful outcomes in disparate organizational settings. Measuring HRF in a systematic and rigorous way would allow us to better understand the extent to which this construct influences individual and team behaviours. Experimental studies that manipulate aspects of HRF and identity leadership would also allow us to examine causal relationships and further investigate the mechanisms through which HRF and identity leadership impact on HRO outcomes.

## CONCLUSION

This study provides an extension to the theoretical understanding of HROs by shedding light on the importance of HRF. We demonstrate that within elite military teams there is evidence for five aspects of HRF, which are underpinned by specific reliability‐related identity content. While the study confirms that HRF principles are present in the teams we studied, our findings suggest that HRF is not simply a mirror image of the HRO principles but rather a textured, nuanced and creative manifestation of them. This means that these high‐reliability principles look different when considered from the viewpoint of the followers who are responsible for ‘doing’ high reliability, and critically they reflect their practical understandings of the shared identity content of the group as it applies to a specific operational context. In this way, high‐reliability followership can be seen as the manifestation of internalized shared social identity content that is made possible by identity leadership and lived out in the practical behaviours of group members on the ground. Ultimately, then, it is this shared identity content—and the leadership that builds and sustains it—that allows the group to ‘do’ high‐reliability. This is because it is this that ensures that doing one's job ‘super well’ is what ‘we are all about’.

## AUTHOR CONTRIBUTIONS

**Sally Knox:** Conceptualization; investigation; funding acquisition; writing – original draft; methodology; writing – review and editing; formal analysis; project administration; validation; visualization; software. **Kïrsten A. Way:** Supervision; writing – review and editing; conceptualization; methodology; formal analysis. **S. Alexander Haslam:** Conceptualization; writing – review and editing; supervision.

## FUNDING INFORMATION

This research was supported by a Sir Richard Williams Foundation Scholarship to the first author.

## CONFLICT OF INTEREST STATEMENT

The authors declare no conflicts of interest.

## Supporting information


Data S1:

## Data Availability

The data that support the findings of this study are available on request from the corresponding author. The data are not publicly available due to privacy or ethical restrictions.
