# Supplementary material for: ‘Whatever your job is, we are all about doing that thing super well’: High‐reliability followership as a key component of operational success in elite air force teams
Source: Br J Soc Psychol. 2025 Apr 1;64(2):e12882. doi: 10.1111/bjso.12882 (PMC11960839; doi:10.1111/bjso.12882)
Supplement: Supplementary file 1 — Data S1: [file BJSO-64-0-s001.docx]

# Supplementary Material S1: Interview Protocol

This study aims to investigate aspects of leadership and followership in small military groups. In order for me to analyse these issues, I’m going to ask you a series of questions about an event on operations you were involved in when you felt you had to make a decision with an ethical component. During the interview, I would really like you to reflect on your own and others leadership and behaviour. Do you recall the event?

Tell me about your job position at the time of the event.

**Prompt Questions if not covered by individual:**

What was your role in the organisation?

What was your seniority at the time?

How long had you been doing the job?

How long had you been on the SQN?

Starting at the beginning of the mission planning, can you tell me, in your own words, what happened? Please describe in as much detail as possible including what you did, who you spoke with, what was said and what you were thinking/feeling leading up to and during the event.

**Prompt Questions if not covered by individual:**

What thoughts were going on in your head at that time? (used throughout interview to prompt more detail)

Where was your focus on at that time? (used throughout interview to prompt more detail)

How did you think of yourself within the command structure?

How would you describe your relationship with (the different levels of leadership, for example the pilot, the EWO/WSO, the Formation Lead, the Package Commander, the Mission Commander)?

Who did you feel responsible to?

Did you consider yourself following orders?

Did you feel an obligation to the leaders?

How did this feed into your actions?

What do you think led you into the event (crisis)?

What got you out of the crisis?

Was there a time you could pinpoint where it came close to going very badly wrong?

Why didn’t it?

*If this is the second interview with the same participant, include the question (as appropriate):*

How did this experience / your role differ from that discussed in the previous interview?

Did you consider anything about the lead up or context of the event to be novel? (If required, give participant a description of a novel situation using VUCA example.)

Can you describe how you perceived it to be novel?

Can you rate the novel-ness of the situation on a scale of 1 to 10?

Can you give me an example, does not need to relate to this event but to give me an understanding, of both a 1 and also a 10 on your scale?

Tell me about the leadership structure at the time of the event?

(If expansion is required: By leadership I mean not only within the aircraft, but also the formation, and the Package/Mission Commander, as well as any other leadership influences you feel were important.)

To what extent where these individuals ‘doing’ leadership?

Who should have been, but wasn’t?

Thinking back to the event, how did you personally contribute to avoiding it?

What did the leaders do to contribute to it not going wrong?

What didn’t they do?

For you and your experience, who do you think was the most significant leader?

Prompt them about whether they display each of the four leader identity types. Describe them in terms of: Representative of the group (prototype); advance the interests of the group (champion); was the group shaped by the leader (entrepreneur); reality interpreted in the image of group identity (impresarios), or a combination of these.

Tell me about your team at the time of the event. (Prompt: How would you describe them as a team to others; what is important to them and what defines them.)

How strongly do you identify with the team?

Can you rate this on a scale of 1 to 10?

Can you give me an example, does not need to relate to this event, of a team or group you identify with as both a 1 and a 10?

Do you think the way you talk about the incident is different now to directly after the event? If so, in what way and why?

Is there anything else that you think might be important, from a leadership or followership perspective, with regards to this event? (If required: By followership I mean your response to the leadership, not only your behaviour but also how you felt about it.)

**If this is the second interview with the same participant, include the question (as appropriate):**

How did the leadership / leader /team differ from that discussed in your previous interview?

Final question…what does good leadership look like?

# Supplementary Material S2: Example of Inductive Coding from the Data Corpus

| **Codes** |
| --- |
| Noticeable behaviour |
| Aggression towards enemy |
| Ambition |
| Justification of actions |
| Looking back on the event |
| Mention of foreigners |
| Mention of the alpha personality |
| Physical positioning |
| Reflections on VUCA |
| Relations with others on squadron |
| How we are better than the ones that came before |
| Good memories |
| This is serious stuff here |
| Micro HRO |

Note: This example reflects a sample of codes generated during data analysis rather than the entire coding structure. Codes in this example were derived from the data through iterative analysis

# Supplementary Material S3: Example of Deductive Coding from the Data Corpus

| **Code** | **Sub-code** |
| --- | --- |
| HRO | Reluctance to Simplify |
|  | Pre-occupation with Failure |
|  | Commitment to Resilience |
|  | Deference to Expertise |
|  | Sensitivity to Operations |
| Shared Identity | What is “us” |
|  | Creation of “us” |
|  | Protecting “us” |
|  | What makes “us” different |
|  | Who “us” is |
|  | Who “us” isn’t |
| Identity Leadership and Followership | Leadership |
|  | Followership behaviour |
|  | Leaderful behaviour |
|  | Un-leaderful behaviour |

Note: This example reflects a sample of codes used during analysis rather than the entire coding structure. Codes in this example were derived from established theoretical concepts and applied systematically to relevant participant accounts.

# Supplementary Material S4: Representative Quotes Illustrating Subthemes of High-Reliability Followership (HRF)

| **Theme** | **Looks like*** | **Representative quotes** |
| --- | --- | --- |
| Engaging with ambiguity | We know that ambiguity and uncertainty are inevitable in our work. | There is always uncertainty in those missions, but I don’t think that’s something that impacts your thought process or your game plan, the uncertainty is certain. [Pilot] |
|  | We seek out uncertainties and take active steps to manage them. | I think ambiguity is the one thing that we do try and really flush out with things like rules of engagement, and other things, codes and checklists and all those types of things. [Pilot] |
|  | We do this even though we know that we won’t have all the solutions. | We prepare ourselves for a volatile, uncertain thing because the enemy always gets the vote in the outcomes. [Pilot] |
|  | We actively make our behaviours predictable when we are faced with unexpected or ambiguous events. | Because you’ve got to know what you’re doing and you’ve got to make sure that you’ve taken that ambiguity and you’ve brought it down to a mission set that everyone understands how they are going to operate. [Pilot] |
| Attending to failure | We feel a strong sense of responsibility to improve. | No mission is perfect, if you go out there and do a perfect mission, you just didn’t find the thing you can improve on effectively. [WSO] |
|  | We are motivated to root out causes of errors. | We just went through the process of trying to have a look, okay, how do we stop this happening again or what else could we have done? [Pilot] |
|  | We are honest when appraising our own roles in anticipating or responding to problems. | In the debrief it was obviously my fault and it was…I found it not hard to admit. It’s disappointing, but I had no sort of compunction with going hey, that was…that was my bad. Standing up in front of the squadron talking about the assumptions I made and etc like that. [Pilot] |
| Perseverance through setbacks | We do not wait for mistakes to happen, and we persistently evaluate what we do to ensure that we are employing best practices in our daily work. | Let’s spend our relatively precious time concentrating on the things that could be done better next time around. [Pilot] |
|  | When things go wrong, we are able to quickly regroup and carry on. | You debrief it and then you move on, you don’t harp on it, you don’t keep looking aback. [Pilot] |
|  | We support each even if actions have led to task failure but only if those actions were consistent with our group identity. | The importance of integrity and also a sense of the importance of having an open and honest debrief where we have an almost attributionless—well attributionless isn’t true—but a no consequence culture of getting out the things that could be improved on. [Pilot] |
| Proffering expertise | We know that expert contribution is valued, sometimes more than organisational hierarchy. | We respect specialisation above rank, and I think that comes back to the basics of even maintaining an aeroplane. If the corporal tells you the aeroplane is not serviceable to fly, you’re not going to argue with them, are you? That’s his specialty [Pilot] |
|  | We actively maintain our expertise and will provide it when required for a task. | He’s exceptional at what he does, always trying to be better, never…even he would admit to coming to someone like me if I had more experience in a certain field to ask questions and stuff and would actively do that with people. [WSO] |
|  | We know the limitations of our expertise and rely on credible subject matter experts when required. | Air Force is about a knowledge economy. So, it is about SME [*subject matter expert*] input being the most valuable contribution... [Pilot] |
| Striving for team success | We understand that our contribution is a critical component of overall team success. | I can’t just kick the can down the road and make it someone else’s problem, so I want to fight the best fight I can today, so the person tomorrow has the best chance to win their fight. [WSO] |
|  | We are clear in our task focus and act to ensure there is excellence in all that we do. | Taking that professionalism very seriously with an overarching understanding of, “the missions we do are important and we are not going to … not get distracted from what our mission is”. [Pilot] |

Note: *The "Looks like" column is an elementised version of the HRF descriptions listed in Table 3: Hallmark practices of HROs and HRF and also represent the second order coding structure
